# Deep Learning for Describing Breast Ultrasound Images with BI-RADS Terms

**DOI:** 10.1007/s10278-024-01155-1

**Published:** 2024-06-26

**Authors:** Mikel Carrilero-Mardones, Manuela Parras-Jurado, Alberto Nogales, Jorge Pérez-Martín, Francisco Javier Díez

**Affiliations:** 1 Department of Artificial Intelligence, Universidad Nacional de Educacion a Distancia (UNED), Madrid, Spain; 2 Department of Radiology, HM Hospitals, Madrid, Spain; 3 CEIEC Research Institute, Universidad Francisco de Vitoria, Madrid, Spain

**Keywords:** Breast ultrasound, BI-RADS, Medical image captioning, Computer-aided diagnosis, Attention mechanisms, Explainable artificial intelligence

## Abstract

Breast cancer is the most common cancer in women. Ultrasound is one of the most used techniques for diagnosis, but an expert in the field is necessary to interpret the test. Computer-aided diagnosis (CAD) systems aim to help physicians during this process. Experts use the Breast Imaging-Reporting and Data System (BI-RADS) to describe tumors according to several features (shape, margin, orientation...) and estimate their malignancy, with a common language. To aid in tumor diagnosis with BI-RADS explanations, this paper presents a deep neural network for tumor detection, description, and classification. An expert radiologist described with BI-RADS terms 749 nodules taken from public datasets. The YOLO detection algorithm is used to obtain Regions of Interest (ROIs), and then a model, based on a multi-class classification architecture, receives as input each ROI and outputs the BI-RADS descriptors, the BI-RADS classification (with 6 categories), and a Boolean classification of malignancy. Six hundred of the nodules were used for 10-fold cross-validation (CV) and 149 for testing. The accuracy of this model was compared with state-of-the-art CNNs for the same task. This model outperforms plain classifiers in the agreement with the expert (Cohen’s kappa), with a mean over the descriptors of 0.58 in CV and 0.64 in testing, while the second best model yielded kappas of 0.55 and 0.59, respectively. Adding YOLO to the model significantly enhances the performance (0.16 in CV and 0.09 in testing). More importantly, training the model with BI-RADS descriptors enables the explainability of the Boolean malignancy classification without reducing accuracy.

## Introduction

Breast cancer is the most frequent type of cancer in women, with approximately 2.3 million cases worldwide in 2022 [1]. The 5-year survival rate is 90% overall; it increases to 99% when cancer is detected in an early stage, i.e., when it is localized,^1^ but reduces to 86% when it has spread to regional lymph nodes and to 29% if it has spread farther. Therefore, early detection is a primary concern. In some low-income countries, the 5-year survival rate is only 20% [2], partly due to the lack of tests and experts necessary to implement appropriate screening programs.

Different techniques exist for diagnosing breast cancer, such as ultrasound, mammography, and magnetic resonance. While mammography has proven to be the most effective breast cancer screening test, it has some disadvantages: the patient is exposed to radiation that may increase the probability of developing cancer [3]; its sensitivity is lower in dense breasts, which are more common in young women [4]; and breast compression causes discomfort and pain [5].

In contrast, ultrasound is non-invasive, painless, and cheaper. It can detect nodules that may go unnoticed in dense breasts and clarify some specific tumor characteristics. However, ultrasound is a low signal-to-noise ratio technique, and the quality of the test depends on the skills of the user. For these reasons, computer-aided diagnosis (CAD) systems improve tumor detection and diagnosis and reduce examination time [6].

With technological advances and the availability of large datasets, artificial intelligence (AI) is helping to solve a wide variety of problems in different areas, such as speech recognition, data mining, natural language processing, or computer vision. Deep learning, in particular, has dramatically improved state-of-the-art results due to its ability to obtain abstract characteristics from the data [7]. Deep Neural Networks (DNN) iteratively learn from the data by adjusting the parameters in their layers via optimization methods, such as stochastic gradient descent. In computer vision, Convolutional Neural Networks (CNNs) have fewer parameters per layer than other DNNs and can learn space-invariant characteristics, thus allowing deeper architectures. Since AlexNet won the Imagenet competition by a significant margin in 2012 [8], most winners of that contest have been CNN-based models. Deep learning models can take too long to learn, but transfer learning (using a model pre-trained on a different data set) can alleviate this problem.

In medicine, many CADs have been developed using pre-trained CNNs [9], such as DenseNet, ResNet [10], or VGG [11]. The main problem of these architectures is that they are black-box models, and physicians are reluctant to accept the advice of a machine if they cannot understand the reason for it. Moreover, the European General Data Protection Regulation (GDPR) establishes that explanations of machine learning techniques used for decision making are mandatory [12]. For these reasons, eXplainable Artificial Intelligence (XAI) techniques have gained interest in recent years, especially in medical imaging. Most of the current work in this field uses visual explanation, while text-based and example-based explanations are less frequent [13]. Visual explanation focuses on where the algorithm has put more attention; it can return, for example, the Regions of Interest (ROIs) where the model has seen malignancy traces. This can help doctors to detect and conduct a biopsy on possible cancers. Nevertheless, these algorithms do not explain why the model has focused on a certain area. In some scenarios, it may confuse physicians and lower the trustworthiness of the model [14].

Most CAD systems classify ultrasound images as normal, benign, or malignant. In particular, Liu et al. [15] combine CNNs with an iterative neighborhood component analysis to select the most important features before introducing them to a DNN, resulting in the best accuracy obtained for a publicly available dataset, $97.18\%$.

Radiologists describe breast tumors using the Breast Imaging-Reporting and Data System (BI-RADS), a standard language that contains several descriptors, such as shape, margin, orientation, echogenicity, and posterior enhancement. They are used to infer a class from the BI-RADS tumor malignancy scale and select an intervention, as shown in Table 1.

Some machine learning models use the descriptors assigned by radiologists as inputs for a Boolean malignancy classification algorithm, with excellent results [16, 17]. Others generate the descriptors from manually segmented images [18, 19] and input them to the algorithm without checking if they are correct or not. Both approaches require a significant effort from experts.

To our knowledge, four studies have focused on measuring the accuracy of the BI-RADS descriptors returned by AI systems from ultrasound images [20–23]. One of them compares Samsung’s S-detect system—which also requires manual segmentation of images—with an expert radiologist [20]; as it is a proprietary software, no information about the algorithm is available. The second uses a synthetic dataset of 4458 images, described by three radiologists, to train deep neural networks with attention mechanisms that infer certain BI-RADS descriptors from the ROI of each nodule [21]. In this case, the only work required from the experts is to extract the ROI. Again, the aim of this study was to obtain the best descriptors to input them into a classification algorithm, not to generate medical reports. Other two conference papers address the generation of breast ultrasound BI-RADS descriptors [22, 23], using a similar architecture. The second also returns the tumor segmentation, but only focuses on Boolean malignancy classification, not BI-RADS classification [23]. This paper offers explanations for this Boolean classification using SHAP values, a method that we explored and discarded, for the reasons given in Section 4.

Finally, Kaplan et al. propose an innovative architecture that returns the BI-RADS malignancy classification with an accuracy of 80.42, using 1038 images from a public dataset and from their own patients [24]. However, the model does not provide the BI-RADS descriptors or any other explanation for the classification.

**Table 1:** BI-RADS classification and subsequent clinical intervention

BI-RADS category	Likelihood of malignancy	Intervention
0	Incomplete	Additional evaluation
1	No findings	Normal procedure
2	Benign	Normal procedure
3	Probably benign ($<2\%$)	Control in 6 months
4A	Low suspicion ($2-10\%$)	Biopsy
4B	Moderate suspicion ($10-50\%$)	Biopsy
4C	High suspicion ($50-90\%$)	Biopsy
5	Probably benign ($>90\%$)	Biopsy
6	Proven malignant by biopsy	Treatment

In this context, we present an innovative CAD system that can aid physicians through the process of detecting, describing, and classifying tumors. It incorporates YOLO as a preprocessing step to detect the ROIs, thus discarding non-essential information and allowing the system to analyze more than one nodule in each image. These ROIs are fed to a multi-class classification network, which outputs the BI-RADS descriptors, the BI-RADS classification, and a Boolean estimation of malignancy. It also yields the echogenic halo characteristic and detects the BI-RADS special cases (for example, complex cysts, simple cysts), from now on known as suggestivity. We prove that this CAD does not lose performance when going through each step; on the contrary, each phase enhances the following ones.

Our study differs from previous work in four aspects. First, our system covers the entire process of breast ultrasound examination, from automatic tumor detection to tumor description and final diagnosis. Second, we detected some duplicates in three public datasets, especially in one of them [25], which had not been reported in previous studies using the same images; we removed them before training and testing our models because they may lead to overestimation of accuracy. Third, our model is able to explain the BI-RADS malignancy classification based on the BI-RADS descriptors, using the weights of a multinomial logistic regression. Finally, the detection of ROIs with YOLO, an algorithm capable of real-time video processing, will allow the integration of our model into a system that can assist sonographers in real time, i.e., while examining the patient.

The rest of the paper is structured as follows: Section 2 presents the data preprocessing, the architecture of our model, and the experiments comparing it with different state-of-the-art CNNs. Section 3 shows the results of the experiments, and Section 4 discusses them. Finally, Section 5 gives a brief summary and proposes lines for future work.

## Methods

### Data Preparation

We have worked with three different public datasets, namely, BCD [25], B [26], and BUSIS [27]. BCD contains 780 images classified as normal (133), benign (487), or malignant (210); B has 163 images, labeled with the tumor type (cyst, fibroadenoma, etc.) and the malignancy classification (110 benign and 53 malignant); and BUSIS has 562 images with no label.

**Fig. 1:**
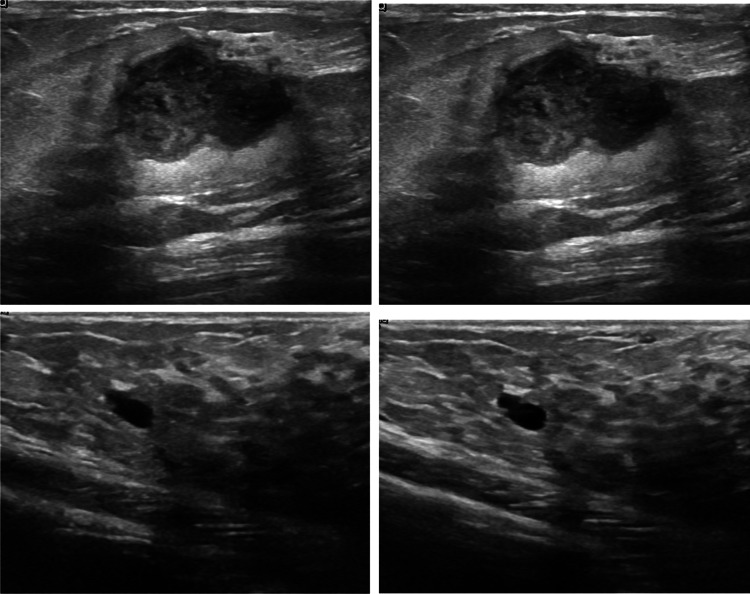
Detection of duplicates. The two images in the upper row are detected by SIFT as copies in the BCD dataset, one was labeled as benign and the other as malignant. The images in the lower row contain the same nodule across time; SIFT does not detect them as duplicates, but we only used one of them for description and classification

Our study revealed some limitations in BCD that are not reported in the literature. First, in images with several nodules, some of them are not segmented, especially simple cysts. Furthermore, using the Space-Invariant Feature Transform (SIFT) algorithm to detect zoomed or rotated copies of the same image [28], it was found that 150 images had at least one almost identical copy, 8 of them in a different class (see Fig. 1): 6 of these were classified as both benign and malignant, and the other 2 as benign and normal. For this reason, 189 images were discarded. SIFT did not detect images corresponding to the same nodule taken at different times during the ultrasound scan (see Fig. 1). We also excluded these images from our dataset. We applied SIFT to the other two datasets, finding 2 duplicate images in B and 8 in BUSIS. After cleaning duplicates and images corresponding to the same tumor, we assumed that the remaining images corresponded to different tumors. Our cleaning helped to avoid having the same or very similar tumors in training and testing, which could generate bias.

Additionally, to avoid the “Clever Hans phenomenon” [29], which consists in producing correct classifications based on “spurious” features, images with extra information, such as color maps or delimited ROIs, were only used for training (because they improved the results), not for validation or testing.

All three databases have numerous simple cysts, which are the easiest to detect and describe; in fact, our model trained with only 75 images (25 of them were simple cysts) was able to detect and describe them correctly. We discarded most of these simple cysts and focused on more complex nodules.

In conclusion, we obtained from these datasets a total of 749 images: 154 from B, 339 from BCD, and 256 from BUSIS, with their corresponding malignancy classification, if available (306 were benign and 177 malignant). Given that these public datasets do not contain BI-RADS descriptors or ROIs, the images were annotated by one of the authors (MPJ), a breast radiologist with more than 30 years of experience. More information about the descriptors we used can be found in Appendix A.

### Architecture

The core of our system is a model consisting of two elements: a multi-class classification network with an attention mechanism that returns the BI-RADS descriptors and the Boolean malignancy classification, and a multinomial logistic regression that returns the BI-RADS classification. It is preceded by a YOLO module that obtains the ROIs and followed by a rule-based model that fine-tunes the results and gives the final output in natural language.

#### Detecting ROIs with YOLO

We use YOLO [30] to detect the ROIs, i.e., the nodules, in each image. Since this is a fully convolutional algorithm, it can take different images of different sizes and shapes as input. YOLO is very fast, and its eighth version, YOLOv8, runs at 50 frames per second. If the width or height of a detected ROI is higher than 450, the image is resized keeping the width-height ratio, which is relevant for some descriptors, such as the orientation. We then use zero-padding to fill the ROIs to 450$\times$450 pixels. This padding does not affect the CNN training procedure and has no negative impact on time performance [31].

#### Extracting BI-RADS Descriptors

**Fig. 2:**
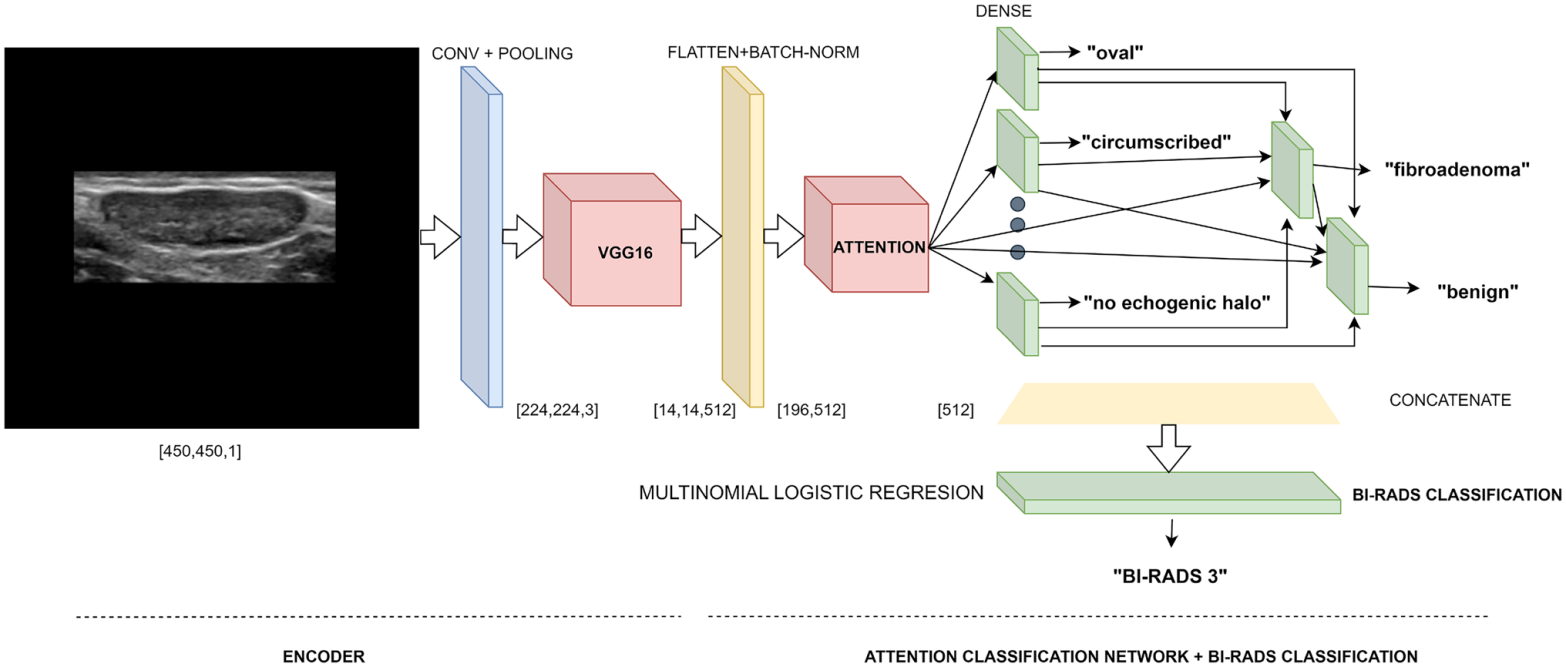
This model receives as input a ROI, passes it through a convolutional layer with max-pooling and a VGG16, which gives the feature space, and normalizes it. Then, the attention classification network returns the tumor descriptors, such as “oval”,“circumscribed”, etc., as well as the type of tumor (“fibroadenoma”) and the Boolean malignancy classification (“benign”). A multinomial logistic regression uses all these features, except the Boolean malignancy classification, to yield the BI-RADS classification

As mentioned above, the core of our system is the model shown in Fig. 2. The first of its two elements is a multi-class classification algorithm, which takes as input each nodule extracted by YOLO and outputs its BI-RADS descriptors and the Boolean malignancy classification. The multi-class classification algorithm has an encoder consisting of a convolutional layer with max-pooling and GELU activation function [32], and a VGG16 [11] with an output size of 14$\times$14$\times$512; i.e., for each image, it yields a 196$\times$512 feature-space matrix, $\textbf{F}$, which is then batch-normalized.

The attention component of the classification network computes a weighted average of the feature space, known as *context* [33], calculated as follows:

1 $$\begin{aligned} \text {\textbf{c}}=\mathbf {a\cdot \textbf{F}}=\sum _{i=1}^{196}a_{i}\cdot \textbf{f}_{i}\;, \end{aligned}$$

where $\textbf{f}_{i}$ (a vector of dimension 512) is the *i*-th row of $\textbf{F}$ and $\textbf{a}$ is a vector of weights, such that

2 $$\begin{aligned} a_{i}=\frac{\exp (\tanh ({\textbf {V}}\cdot \textbf{f}_{i}))}{\exp (\sum _{j=1}^{196}\tanh ({\textbf {V}}\cdot \textbf{f}_{j}))}\;. \end{aligned}$$

**V** is a weight matrix learned during training. This means that we first input each feature-space vector $\textbf{f}_{i}$ into a perceptron with a hyperbolic tangent (tanh) activation function that returns its “importance” in a range from $-1$ to 1. We then concatenate all of them into a softmax activation function that gives an ordered output of these “importances,” $a_{i}$, ranging from 0 to 1, which add up to a total of 1, so that we can calculate the weighted average of the feature space. Since the $\textbf{f}_{i}$’s are the output of the convolutional encoder and we calculated the “importance” of each one, $a_{i}$, these weights indicate the regions of the image to which the model has paid more attention.

This context, $\textbf{c}$, is the input to six dense layers, one for each BI-RADS descriptor: shape, margin, orientation, echogenicity, posterior features, and halo. Each layer has one output neuron for each possible value of the descriptor, with a sigmoidal activation function for orientation, and a softmax for the other descriptors. The sigmoidal function was chosen because some nodules are neither parallel nor anti-parallel (for example, round tumors); therefore, our model only provides this descriptor when the result of the sigmoid function exceeds a certain threshold, empirically set to 0.3.

The dense layer that gives the suggestivity or tumor type, which in Fig. 2 returns the label “fibroadenoma,” receives the output from the layers of the six BI-RADS descriptors (because the suggestivity of a tumor depends on them) and the context, $\textbf{c}$. We assigned to this layer a softmax activation function and an extra label, “no clear suggestivity,” because for 484 of the 749 nodules, the radiologist could not choose a value for this descriptor—a sigmoid activation function would have returned a label for those nodules, regardless of the threshold.

Finally, the dense layer for the Boolean malignancy classification (which in Fig. 2 returns the label “benign”) receives the descriptors, the context, and the suggestivity as inputs and combines then with a softmax.

#### BI-RADS Multinomial Logistic Regression

**Fig. 3:**
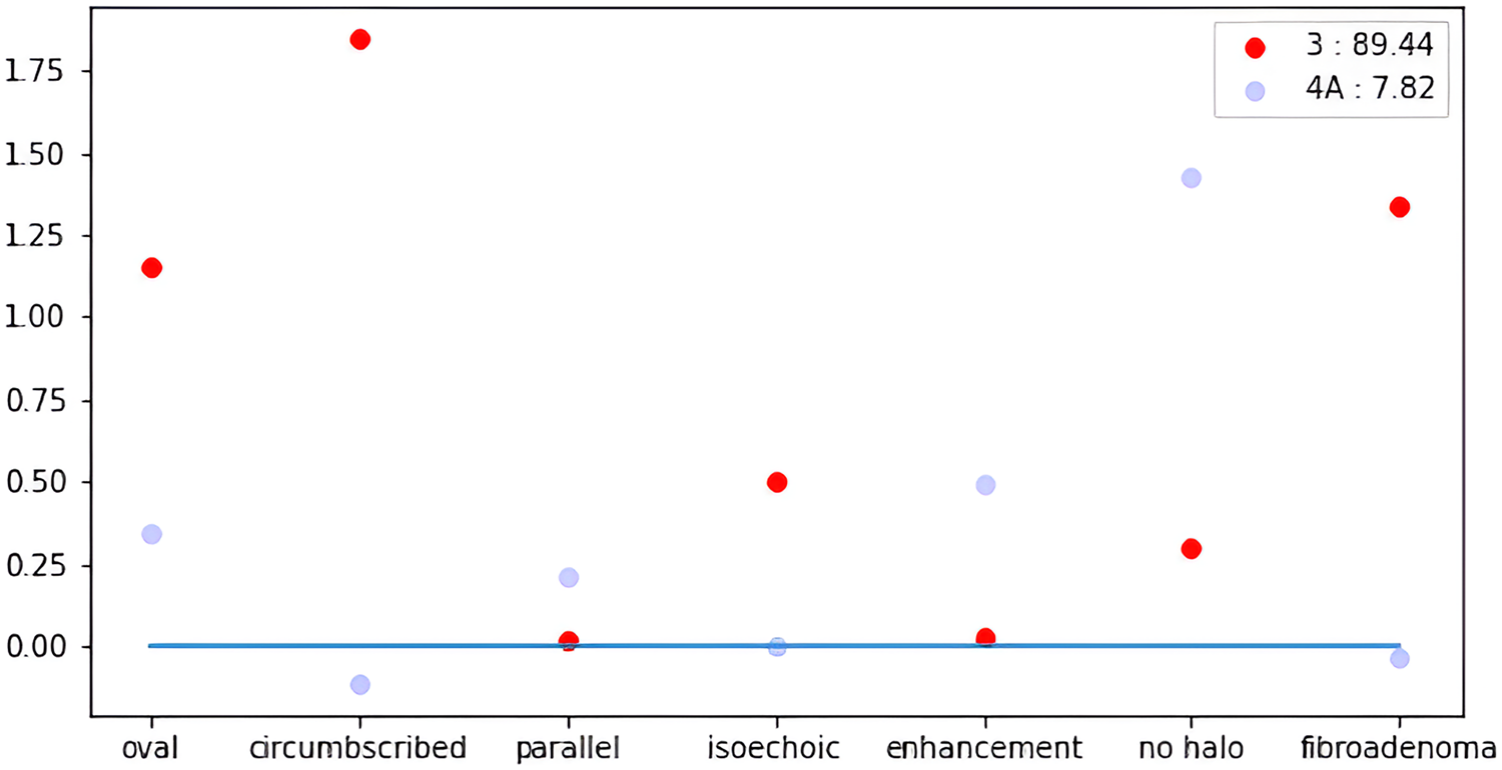
Descriptors’ weights for the example in Fig. 2. Red points are the weights of BI-RADS 3 output, while blue points are the weights of the second option BI-RADS 4A

The second part of the model is a multinomial logistic regression that receives the descriptors and outputs the nodule final BI-RADS classification (the label “BI-RADS 3” in Fig. 2). We did not incorporate this into the multi-class classification model because it worsened the performance. In addition, basing the output only on the descriptors and not on the image itself allows the system to explain the classification. One of the benefits of using a multinomial logistic regression is the simplicity and explainability of the algorithm. For example, Fig. 3 shows the “importances”/weights of the descriptors for BI-RADS 3 and 4A for the example in Fig. 2.

#### Generating Natural Language Descriptions with a Rule-Based Module

**Fig. 4:**
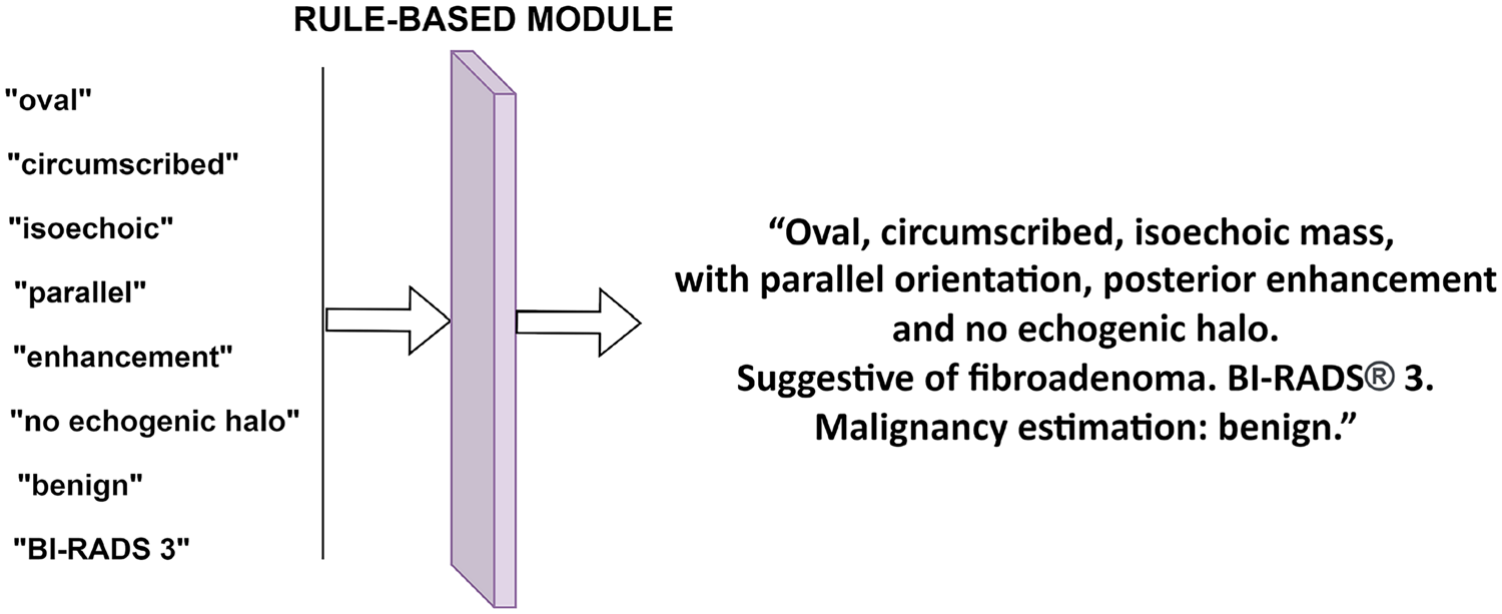
A rule-based module fine-tunes the output and generates natural language descriptions

Finally, two rules taken from the latest edition of the BI-RADS standard [34] are applied to fine-tune the output: (1) if the nodule is round, it has no orientation, neither parallel nor anti-parallel; (2) when the nodule is classified as a simple cyst, a complex cyst, or is spiculated, the BI-RADS classification is set to 2, 4A, and 5, respectively. The first rule applies to the orientation output of the multi-class classification network, eliminating its output. The second rule only applies to the BI-RADS multinomial logistic regression model. Additional rules are used to generate a natural language description, as shown in Fig. 4, but do not modify the model results.

### Experiments

#### Detecting Nodules with YOLO

We first tested the ability of the YOLO’s preprocessing module to detect nodules. We randomly selected 600 of the 749 nodules and used the images for training, augmenting them with YOLOv8’s facilities, which consist of random scaling, color space augmentations, and mosaic data loader (merging more than one image into one); and 149 nodules for testing. When training, the images were resized to 480$\times$480 pixels; YOLOv8 automatically applies zero-padding to maintain the image height-width ratio. We analyzed whether the performance of our multi-class classification network decreases when taking as input the ROIs trimmed by the YOLO module instead of those segmented manually.

#### Describing and Classifying the Nodules

We compared the BI-RADS descriptors and the BI-RADS classification from our model with those of the expert and the Boolean malignancy classification from our model with the ground truth recorded in the B and BCD datasets. We also compared with this ground truth our expert’s Boolean malignancy classification (considering tumors with BI-RADS 4A or lower as benign and the others as malignant). We analyzed the impact of each component of the system on the performance; in particular, we studied different versions of the system:

1. A model consisting only of a plain classifier pre-trained on ImageNet (namely VGG16, ResNet [10], DenseNet  [35], MobileNet [36],
2. The residual attention network [37] used as base for the model in [21] and pre-trained on ImageNet,
3. A model that only has the attention layer and the Boolean malignancy classification layer (the one that outputs the label “benign” in Fig. 2); i.e., it dispenses with all the other layers in the classification network so that the output of the attention mechanism is the only input of the Boolean malignancy classification layer, and
4. Our model receiving the images directly, i.e., without preprocessing them with the YOLO detector.

We also tried out the pre-trained DenseNet model on the RadImageNet dataset [38], which we named RAD-DenseNet. More information about the architectures and hyperparameters of all models can be found in Appendix B.

Our experiments also tested VGG19 as an alternative to VGG16. The results were usually slightly worse, but the difference was very small.

We have performed two series of experiments. In the first one, we did two repetitions of 10-fold cross-validation with the 600 manually selected ROIs used to train YOLO. One of the folds was never used for validation, since it contained images with extra-information, such as color maps, delimited ROIs, and tumor segmentation. We augmented the images with

- Random enlargement or reduction of the ROI size in the original image: $[-0.1:0.25]$ times the size of the ROI vertically and $[-0.1:0.15]$ horizontally.
- Random zoom of the ROI: $[-0.3:0.3]$ times the size of the ROI.
- Random contrast and brightness alterations: [0.8 : 1.2] contrast and $[-25:25]$ brightness alterations.
- Random horizontal flips.
- Random rotations, limited to a maximum range of $0.05*2\pi$ radians to preserve tumor orientation.

In the second series of experiments, called “testing,” we used the 149 ROIs detected by YOLO and repeated the experiment 5 times.

We recall that for the BI-RADS category classification, we used the same multinomial logistic regression function, trained with the descriptors and categories given by our expert. Therefore, the weights of the algorithm were the same for all the models. The results in validation and test of the BI-RADS category will indicate the quality of the combination of the descriptors given by these models and will thus also assess their performance.

## Results

### Detecting Nodules with YOLO

**Fig. 5:**
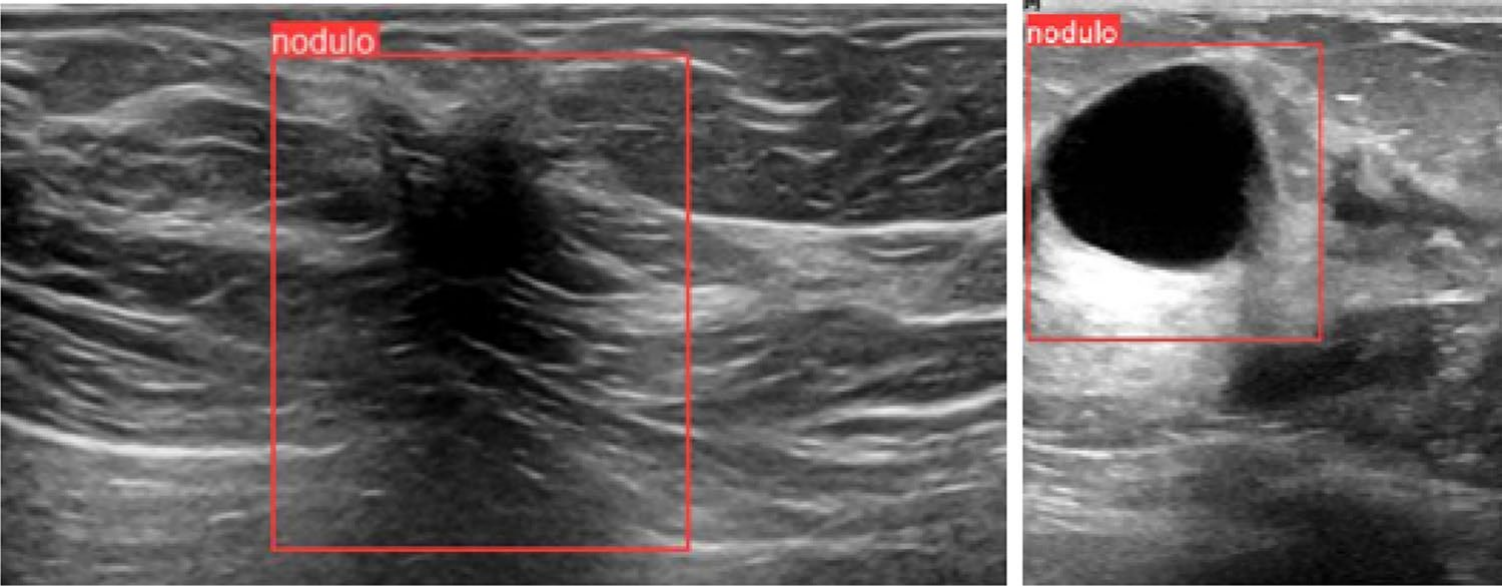
YOLO captures the part of the image necessary to extract the posterior feature; for example, for a malignant tumor with posterior shadowing (left) and for a cyst with posterior enhancement (right)

The YOLO module for detecting nodules obtained a precision of 0.93, a recall of 0.95, and an average precision (AP) of 0.97. Figure 5 shows that YOLO can also capture the part of the image necessary to extract the posterior feature. The threshold with which the algorithm detects the nodules can be changed by the radiologist for each image, to include a nodule that YOLO has skipped, or vice versa. To obtain the ROIs of the nodules that YOLO did not detect in the test, we lowered the threshold. This way, we could test the description and classification models with the ROIs obtained by YOLO rather than manually.

**Table 2:** Agreement on BI-RADS descriptors (Cohen’s kappa). The first three rows, given as a baseline for comparison, are obtained from the literature. The others compare different models with the values assigned by our expert. The labels “orient.”, “echog.”, and “sugges.” are abbreviations for orientation, echogenicity, and suggestivity

Model	Shape	Margin	Orient.	Echog.	Posterior	Halo	Sugges.	**Mean**
intercorrelation	$0.48\pm 0.10$	$0.34\pm 0.04$	$0.60\pm 0.02$	$0.35\pm 0.04$	$0.50\pm 0.05$	$0.58\pm 0.06$	-	$0.47\pm 0.11$
intracorrelation	$0.68\pm 0.06$	$0.59\pm 0.04$	$0.76\pm 0.05$	$0.72\pm 0.06$	$0.69\pm 0.06$	$0.72\pm 0.10$	-	$0.70\pm 0.03$
Samsung	0.64	0.30	0.61	0.34	0.29	0.26	-	0.41

**Cross-validation**								
VGG16	$0.54\pm 0.09$	$0.50\pm 0.07$	$0.65\pm 0.16$	$0.52\pm 0.05$	${\textbf {0.54}}\pm 0.10$	$0.64\pm 0.07$	$0.60\pm 0.20$	$0.55\pm 0.05$
ResNet	$0.50\pm 0.09$	$0.48\pm 0.10$	$0.64\pm 0.12$	$0.49\pm 0.09$	$0.52\pm 0.10$	$0.66\pm 0.10$	$0.56\pm 0.20$	$0.54\pm 0.10$
DenseNet	$0.48\pm 0.07$	$0.46\pm 0.08$	$0.63\pm 0.10$	$0.46\pm 0.09$	$0.48\pm 0.09$	$0.66\pm 0.08$	$0.55\pm 0.17$	$0.52\pm 0.07$
RAD-DenseNet	$0.48\pm 0.09$	$0.47\pm 0.09$	$0.64\pm 0.13$	$0.42\pm 0.11$	$0.48\pm 0.12$	$0.62\pm 0.09$	$0.48\pm 0.23$	$0.49\pm 0.08$
MobileNet	$0.49\pm 0.08$	$0.46\pm 0.10$	$0.61\pm 0.15$	$0.46\pm 0.08$	$0.48\pm 0.09$	$0.64\pm 0.10$	$0.51\pm 0.22$	$0.51\pm 0.06$
Residual attention	$0.50\pm 0.07$	$0.48\pm 0.08$	${\textbf {0.68}}\pm 0.15$	$0.49\pm 0.08$	$0.51\pm 0.09$	$0.60\pm 0.10$	$0.60\pm 0.18$	$0.54\pm 0.04$
Our model	${\textbf {0.57}}\pm 0.09$	${\textbf {0.54}}\pm 0.07$	$0.62\pm 0.16$	${\textbf {0.54}}\pm 0.05$	$0.52\pm 0.10$	${\textbf {0.67}}\pm 0.07$	${\textbf {0.66}}\pm 0.21$	${\textbf {0.58}}\pm 0.05$
No YOLO	$0.43\pm 0.13$	$0.38\pm 0.12$	$0.46\pm 0.19$	$0.39\pm 0.13$	$0.38\pm 0.13$	$0.54\pm 0.14$	$0.38\pm 0.27$	$0.42\pm 0.11$

**Testing**								
VGG16	$0.57\pm 0.03$	$0.50\pm 0.03$	$0.62\pm 0.03$	$0.54\pm 0.05$	$0.52\pm 0.07$	${\textbf {0.75}}\pm 0.03$	$0.62\pm 0.04$	$0.59\pm 0.01$
ResNet	$0.56\pm 0.06$	$0.48\pm 0.03$	$0.63\pm 0.04$	$0.53\pm 0.05$	$0.55\pm 0.03$	$0.65\pm 0.05$	$0.62\pm 0.04$	$0.57\pm 0.02$
DenseNet	$0.52\pm 0.04$	$0.45\pm 0.02$	${\textbf {0.65}}\pm 0.04$	$0.50\pm 0.07$	$0.51\pm 0.10$	$0.67\pm 0.03$	$0.59\pm 0.01$	$0.56\pm 0.02$
RAD-DenseNet	$0.54\pm 0.03$	$0.46\pm 0.05$	$0.47\pm 0.08$	$0.47\pm 0.05$	$0.53\pm 0.06$	$0.64\pm 0.06$	$0.43\pm 0.03$	$0.51\pm 0.02$
MobileNet	$0.53\pm 0.04$	$0.48\pm 0.02$	$0.54\pm 0.04$	$0.49\pm 0.02$	$0.49\pm 0.07$	$0.65\pm 0.01$	$0.57\pm 0.02$	$0.54\pm 0.01$
Residual attention	$0.53\pm 0.03$	$0.45\pm 0.03$	${\textbf {0.65}}\pm 0.04$	$0.50\pm 0.05$	$0.57\pm 0.04$	$0.63\pm 0.07$	$0.69\pm 0.03$	$0.57\pm 0.02$
Our model	${\textbf {0.64}}\pm 0.02$	${\textbf {0.58}}\pm 0.02$	$0.60\pm 0.02$	${\textbf {0.59}}\pm 0.02$	${\textbf {0.61}}\pm 0.03$	$0.71\pm 0.01$	${\textbf {0.73}}\pm 0.05$	${\textbf {0.64}}\pm 0.01$
No YOLO	$0.54\pm 0.02$	$0.51\pm 0.01$	$0.38\pm 0.02$	$0.49\pm 0.02$	$0.49\pm 0.02$	${\textbf {0.75}}\pm 0.02$	$0.68\pm 0.02$	$0.55\pm 0.01$

### Descriptors and BI-RADS Classification

When a tumor is described by different experts or models, the labels assigned may differ. Table 2 shows the Cohen’s kappas obtained in several experiments—values above 0.8 are considered almost perfect agreement and those between 0.6 and 0.8 substantial agreement. The first two rows present, as a baseline for comparison, the average of the values obtained in previous breast ultrasound studies [39–41] for intercorrelation (agreement between experts) and for intracorrelation (agreement of an expert with him/herself when examining the same images some time later); in fact, the average weighted by the number of tumors in each study. The third row shows the kappas obtained when comparing Samsung’s S-detect software [20] with one radiologist.

The other rows show the results of comparing with our radiologist the different models presented in Section 2.3. Table 4 in Appendix C shows the results for the accuracy metric, and Tables 6, 7, 8, 9, 10, 11, and 12 show the confusion matrix for every descriptor. We can observe that our model obtains best or close to best results for all descriptors in cross-validation and testing and higher mean in both. The VGG16 network is second best in results, which means that the encoder choice for our model was the correct one. The changes we made to VGG16 via the attention mechanism and the connections in the classification network (see Fig. 2) increase the kappa from 0.55 to 0.58 in cross-validation and from 0.59 to 0.64 in testing. The YOLO preprocessing step increases the kappa from 0.42 to 0.58 in cross-validation and from 0.55 to 0.64 in testing.

With respect to the BI-RADS classification (the label “BI-RADS 3” in Fig. 2), the comparison of the category obtained with the multinomial logistic regression from the descriptors of our model with the radiologist returned a kappa of 0.53 in cross-validation and 0.52 in testing, which is higher than the results of the other models, that range from 0.42 to 0.45 in cross-validation and from 0.45 to 0.49 in testing. The results are shown in Table 5 and the confusion matrix with the expert in Table 13.

The agreement of this network with our radiologist is higher than the intercorrelation between experts in [39–41] and also higher than the agreement between Samsung’s S-detect and the radiologist who evaluated it [20].

**Table 3:** Comparison of the Boolean malignancy classification by several models and by an expert with the ground truth indicated in the BCD and B datasets

Model	Accuracy	Recall	Precision	F1	Specificity
**Cross-validation**					
VGG16	$0.88\pm 0.08$	$0.86\pm 0.09$	$0.84\pm 0.13$	$0.85\pm 0.10$	${\textbf {0.90}}\pm 0.09$
ResNet	$0.87\pm 0.06$	$0.85\pm 0.10$	$0.82\pm 0.10$	$0.84\pm 0.08$	$0.88\pm 0.07$
DenseNet	$0.88\pm 0.07$	$0.86\pm 0.09$	$0.82\pm 0.12$	$0.84\pm 0.09$	$0.88\pm 0.09$
RAD-DenseNet	$0.87\pm 0.06$	$0.76\pm 0.11$	${\textbf {0.87}}\pm 0.11$	$0.82\pm 0.09$	$0.93\pm 0.07$
MobileNet	$0.86\pm 0.05$	$0.86\pm 0.10$	$0.80\pm 0.12$	$0.82\pm 0.07$	$0.86\pm 0.09$
Residual attention	$0.88\pm 0.05$	$0.88\pm 0.10$	$0.82\pm 0.09$	$0.85\pm 0.07$	$0.88\pm 0.07$
Our model	$0.88\pm 0.05$	$0.84\pm 0.08$	$0.85\pm 0.09$	$0.84\pm 0.07$	${\textbf {0.90}}\pm 0.06$
No YOLO	$0.74\pm 0.12$	${\textbf {0.90}}\pm 0.11$	$0.64\pm 0.15$	$0.74\pm 0.10$	$0.63\pm 0.21$
Model without descriptors	$0.88\pm 0.05$	$0.86\pm 0.09$	$0.82\pm 0.10$	$0.84\pm 0.07$	$0.89\pm 0.06$
**Expert radiologist**	${\textbf {0.90}}\pm 0.04$	${\textbf {0.86}}\pm 0.08$	${\textbf {0.88}}\pm 0.08$	${\textbf {0.87}}\pm 0.06$	${\textbf {0.93}}\pm 0.06$

**Testing**					
VGG16	$0.89\pm 0.01$	$0.86\pm 0.03$	$0.85\pm 0.01$	$0.85\pm 0.02$	$0.91\pm 0.01$
ResNet	$0.88\pm 0.02$	$0.83\pm 0.06$	$0.84\pm 0.03$	$0.83\pm 0.03$	$0.90\pm 0.02$
DenseNet	$0.89\pm 0.02$	$0.88\pm 0.03$	$0.83\pm 0.05$	$0.85\pm 0.03$	$0.89\pm 0.03$
RAD-DenseNet	$0.87\pm 0.02$	$0.80\pm 0.05$	$0.85\pm 0.04$	$0.82\pm 0.03$	$0.91\pm 0.03$
MobileNet	$0.87\pm 0.01$	$0.86\pm 0.06$	$0.79\pm 0.03$	$0.83\pm 0.02$	$0.87\pm 0.03$
Residual attention	$0.88\pm 0.04$	$0.88\pm 0.01$	$0.82\pm 0.07$	$0.85\pm 0.04$	$0.88\pm 0.06$
Our model	${\textbf {0.92}}\pm 0.01$	$0.87\pm 0.01$	${\textbf {0.90}}\pm 0.03$	${\textbf {0.89}}\pm 0.01$	${\textbf {0.94}}\pm 0.02$
No YOLO	$0.87\pm 0.01$	$0.87\pm 0.02$	$0.83\pm 0.02$	$0.85\pm 0.02$	$0.88\pm 0.01$
Model without descriptors	${\textbf {0.92}}\pm 0.02$	${\textbf {0.92}}\pm 0.05$	$0.87\pm 0.06$	${\textbf {0.89}}\pm 0.01$	$0.92\pm 0.06$
**Expert radiologist**	${\textbf {0.94}}$	${\textbf {0.86}}$	${\textbf {0.97}}$	${\textbf {0.91}}$	${\textbf {0.98}}$

The results for Boolean malignancy classification—in which the ground truth is obtained from the BCD and the B datasets—are similar for all those models in cross-validation, but our model is superior in testing, as shown in Table 3. The model with only the attention layer and the Boolean malignancy classification layer, mentioned in Section 2.3 and referred to as “model without descriptors” in Table 3, has the same accuracy in cross-validation and testing as the full model shown in Fig. 2, but is more sensitive and less specific. We statistically analyzed these results by MANOVA with Pillai’s trace test [42] and $\alpha =0.05$. While there is no significant difference between the models (without considering the “no YOLO” model) for cross-validation, with $p=0.1174$, a significant difference was obtained for testing, with $p=0.0078$. Tukey post-hoc tests [43] were performed to detect where the differences lay; the results showed that our model and the “model without descriptors” yield significant differences for the accuracy and the F1 metric compared to MobileNet, RAD-DenseNet, residual attention and ResNet. The *p*-values can be found in Table 14 in Appendix C.

Training our model for each batch of 20 images cost 0.045 s, using a single NVIDIA V100 GPU with 16 GB of HBM2 memory. The inference time for a single image with this GPU is 0.0025 s and 0.075 with an Intel Xeon Silver 4210 CPU.

## Discussion

We have developed a complete CAD system for breast cancer ultrasound. The use of YOLO as a preprocessing step to obtain the ROIs of nodules reduces non-essential information. Our experiments showed that it can not only detect tumors but also capture their surrounding characteristics, namely, the halo and the posterior feature; thus, the gain from trimming the input is greater than the potential loss of information. This approach differs from previous work using either whole images or ROIs trimmed by human experts, which requires an additional effort.

These ROIs are passed to a multi-class classification model with an attention mechanism that describes in BI-RADS terms, and these to a final multinomial logistic regression to give the BI-RADS category. With 600 images for cross-validation and 149 for testing, our model achieved an agreement with our expert (Cohen’s kappa) higher than the correlation between experts found in the literature and lower than the intracorrelation (of each expert with him/herself after some time) [39–41]. The agreement between our system and the expert is higher than between Samsung’s commercial system S-detect and the expert that evaluated it [20]. We compared this model with other CNNs, yielding better results in both cross-validation and testing. The multinomial logistic regression, which gives the BI-RADS classification based on the descriptors, also showed that our model picks descriptors that match with the final result, even if they are not the same as our expert’s descriptors.

In our model, the Boolean malignancy classification is based on both the image and the BI-RADS descriptors extracted from it. While this model performs similarly to the one based only on images, it can use the descriptors to explain the classification, which is an additional advantage. If there is a contradiction in the output—for example, if an apparently benign tumor described as BI-RADS 2 or BI-RADS 3 is classified as malignant—the radiologist might be advised to carefully examine the image. The multinomial logistic regression, which generates example-based explanations, may help the expert understand the BI-RADS classification.

Many studies have applied artificial intelligence to breast ultrasound using the descriptors generated by human experts to feed Boolean malignancy classifiers. Other systems are able to automatically extract the descriptors from images. We have found only two papers, published recently, that address both tasks [22, 23]. Like our system, they use VGG16 as an encoder and a dense model for BI-RADS descriptor classification, trained with the BCD and BUSIS datasets (among HMSS^2^ in the second article [23]). They demonstrate that training the model with the BI-RADS descriptors enhances the Boolean malignancy classification. However, the authors did not clean the datasets to eliminate duplicated nodules, which are very common in these datasets (see Section 2.1) and manually selected the ROIs. Their architecture is similar to one we had for the plain VGG16 classifier, which, in our experiments, performed worse than the version we have used. In the first article [22], the feature space has dimension 28.058, which they reduce with two dense layers before inputting it to the classification network. In contrast, our attention mechanism generates a context of dimension 512. We changed these layers for an average pooling in our plain classifier, since it also obtained better results. Additionally, both studies convert gray images into 3-channel images by applying histogram equalization and smoothing, and concatenating them. With our data and architecture, this technique made no difference in the results, so we decided to save time by working with gray images directly instead of applying that technique. Moreover, evaluating their results, they only measure accuracy, which is not appropriate when a class predominates by far, as in case of orientation, because most tumors are parallel.

Finally, only the first paper [22] calculates the BI-RADS classification, which is based on both the images and the descriptors. We explored this line, but the images sometimes led to a classification that disagreed with the descriptors, which made our expert distrust the system’s advice. The second paper uses SHAP values to explain the Boolean malignancy classification [23]. We also explored this, but the results were not convincing to our expert. In contrast, a multinomial logistic regression model, which is far simpler, obtained the same results with better explainability.

We want to emphasize the problem of duplicates in publicly available datasets, especially in [25], for which we did not find any mention in the literature. We propose the use of SIFT to remove these duplicates. Some of the works mentioned in this article have used this dataset in their research [15, 22–24]. Only one of them takes a subset of it [24], but they do not mention if they do any cleaning.

The main limitation of our study is that our dataset has only 749 tumors. For this reason, we could not consider every characteristic of the BI-RADS system, since there was not enough data to train the models; therefore, “mixed posterior,” “complex cystic and solid echogenicity,” and calcifications were discarded. In addition, although we performed a conscientious cleanup, the dataset used in our experiments might still contain repeated images of the same tumor. Another limitation is that experts sometimes disagree in the interpretation of breast ultrasound images, so it would have been desirable to involve more radiologists. Finally, we have focused on obtaining the BI-RADS descriptors from the image, but more research should be done on the multinomial logistic regression model we used for the BI-RADS classification.

## Conclusions

We have presented a complete CAD system that can detect nodules in real time, avoiding the need of manual segmentation; when a nodule is found, the system generates the BI-RADS descriptors, the BI-RADS classification (which can be explained with the weights of the logistic regression), and a Boolean malignancy classification and combines them in a natural language report, thus alleviating the expert’s workload in every step of tumor diagnosis. The system can also be useful for training novice radiologists and students. We also intend to implant it in countries lacking experts in breast ultrasound.

Future work includes adding a segmentation algorithm to our system to improve the determination of tumor shape, margin, and orientation. Based on recent works, we will also develop different attention models for subsets of BI-RADS descriptors. We are currently obtaining anonymized images from three hospitals in Madrid, with the corresponding reports, which means that we will be able to train our models with more data from more radiologists. Some innovative and recent architectures have obtained good results for BI-RADS classification and Boolean malignancy estimation [15, 24]; we will analyze their potential to give the BI-RADS descriptors. Finally, we will work on the last part of our model, which computes the BI-RADS malignancy from the descriptors, by comparing different classifiers in terms of performance and explainability.

## Appendix A. BI-RADS Descriptors

We have a total of 23 BI-RADS characteristics given by 7 descriptors:

- Shape (4): oval, round, irregular, or lobulated.
- Margin (5): circumscribed, non-defined, spiculated, angulated, or microlobulated.
- Orientation (2): parallel or antiparallel.
- Posterior characteristic (3): enhancement, shadowing, or no posterior changes.
- Echogenicity (4): anechoic, hypoechoic, isoechoic or heterogeneous.
- Echogenic halo (2): no halo or halo.
- Suggestivity (3): simple cyst, complex cyst, or fibroadenoma.

Although lobulated and echogenic halo were discarded in the last edition of BI-RADS, our expert still uses them for BI-RADS classification. In addition, as there were 60 tumors suggestive of fibroadenoma for our expert, we also added this tumor type to the suggestivity, although it is not considered a special case in the BI-RADS system. There is a possibility that a nodule may have more than one margin, e.g., non-defined and spiculated. In these cases, our radiologist only gave the one that implied the highest probability of malignancy and, therefore, the one that most influenced the BI-RADS classification.

## Appendix B. Architectures and Hyperparameters

In all the plain classifiers described in Section 2.3, we did average pooling of the feature space and replaced the network head with a classification layer for each descriptor. Average pooling was preferred to flattening the feature space based on the results. In all the models, we used a convolutional layer with max pooling and GELU activation function to convert the images to the input dimension of the classifiers (224 $\times$ 224 $\times$ 3). We tested DenseNet121, ResNet18, and the residual attention-56 network. We used the Adam optimization algorithm and trained the models for 70 epochs during cross-validation and testing.

We applied a layer-wise learning rate in all the architectures. The dense layers had a learning rate of 0.001, the first convolutional layer 0.0001, the normalization layers 0.00001, and finally, we decreased the learning rate of the remaining convolutional layers as we went deeper into the network, starting with a learning rate of 0.00005. Our model decreased the learning rate by 0.7 for each convolutional layer: $lr_{i}=lr_{i-1}*0.7$. Since some of the architectures are deeper than others—our model has 13 convolutional layers, while DenseNet has 117—we used the following steps to maintain a similar learning rate for all of them. We divided the number of layers in the deep architecture by the number of layers in our model, let us call it *n*,—$n=117/13=9$—. We could then decrease the learning rate every *n* layers, but we preferred to decrease it in a more continuous way, so we used the following formula: $lr_{i}=lr_{i-1}-(1-0.7)/n*lr_{step}$, where $lr_{i}$ is the learning rate for convolutional layer *i*, which is one deeper than $i-1$, and $lr_{step}$ is updated every *n* layers to be $lr_{i}$. With this formula, we are simply dividing *n* times the difference between the learning rate for two continuous convolutional layers in our model and extracting this value at each intermediate layer in the deeper model.

There are two exceptions to these rules: we trained DenseNet for 100 epochs, as it needed more time to converge, and we assigned an initial learning rate of 0.0001 to the RAD-DenseNet.

We set these architectures and hyperparameters during cross-validation empirically.

## Appendix C. Additional Results

**Table 4:** Agreement on BI-RADS descriptors (accuracy). We compare different models with the values assigned by our expert. The labels “orient.”, “echog.”, and “sugges.” are abbreviations for orientation, echogenicity, and suggestivity

Model	Shape	Margin	Orient.	Echog.	Posterior	Halo	Sugges.	**Mean**
**Cross-validation**								
VGG16	$0.70\pm 0.06$	$0.70\pm 0.06$	$0.86\pm 0.05$	$0.68\pm 0.04$	${\textbf {0.76}}\pm 0.07$	$0.86\pm 0.03$	$0.62\pm 0.12$	$0.74\pm 0.02$
ResNet	$0.67\pm 0.05$	$0.68\pm 0.06$	$0.84\pm 0.06$	$0.66\pm 0.06$	$0.74\pm 0.07$	$0.86\pm 0.03$	$0.61\pm 0.15$	$0.72\pm 0.03$
DenseNet	$0.66\pm 0.06$	$0.68\pm 0.06$	$0.84\pm 0.04$	$0.65\pm 0.05$	$0.70\pm 0.05$	${\textbf {0.87}}\pm 0.03$	$0.60\pm 0.12$	$0.71\pm 0.03$
RAD-DenseNet	$0.66\pm 0.06$	$0.70\pm 0.06$	$0.86\pm 0.05$	$0.61\pm 0.06$	$0.72\pm 0.07$	$0.84\pm 0.03$	$0.58\pm 0.14$	$0.71\pm 0.03$
MobileNet	$0.67\pm 0.05$	$0.68\pm 0.07$	$0.83\pm 0.04$	$0.64\pm 0.05$	$0.72\pm 0.05$	$0.86\pm 0.05$	$0.56\pm 0.16$	$0.71\pm 0.03$
Residual attention	$0.68\pm 0.06$	$0.67\pm 0.05$	$0.86\pm 0.07$	$0.66\pm 0.04$	${\textbf {0.74}}\pm 0.05$	$0.84\pm 0.04$	$0.62\pm 0.12$	$0.72\pm 0.03$
Our model	${\textbf {0.73}}\pm 0.06$	${\textbf {0.73}}\pm 0.05$	${\textbf {0.88}}\pm 0.05$	${\textbf {0.71}}\pm 0.05$	$0.74\pm 0.06$	$0.87\pm 0.05$	${\textbf {0.65}}\pm 0.14$	${\textbf {0.76}}\pm 0.03$
No YOLO	$0.66\pm 0.08$	$0.61\pm 0.08$	$0.80\pm 0.06$	$0.61\pm 0.07$	$0.63\pm 0.08$	$0.78\pm 0.08$	$0.38\pm 0.21$	$0.64\pm 0.06$

**Testing**								
VGG16	$0.73\pm 0.02$	$0.71\pm 0.03$	$0.84\pm 0.04$	$0.70\pm 0.03$	$0.76\pm 0.04$	${\textbf {0.90}}\pm 0.01$	$0.61\pm 0.04$	$0.75\pm 0.01$
ResNet	$0.71\pm 0.04$	$0.69\pm 0.02$	$0.84\pm 0.04$	$0.68\pm 0.03$	$0.77\pm 0.01$	$0.87\pm 0.02$	$0.60\pm 0.03$	$0.74\pm 0.02$
DenseNet	$0.71\pm 0.03$	$0.67\pm 0.02$	$0.83\pm 0.02$	$0.66\pm 0.05$	$0.74\pm 0.05$	$0.87\pm 0.02$	$0.61\pm 0.03$	$0.73\pm 0.01$
RAD-DenseNet	$0.73\pm 0.01$	$0.70\pm 0.03$	${\textbf {0.86}}\pm 0.02$	$0.65\pm 0.03$	$0.77\pm 0.03$	$0.86\pm 0.02$	$0.53\pm 0.07$	$0.73\pm 0.02$
MobileNet	$0.70\pm 0.02$	$0.69\pm 0.01$	$0.78\pm 0.02$	$0.66\pm 0.02$	$0.75\pm 0.03$	$0.86\pm 0.00$	$0.61\pm 0.02$	$0.72\pm 0.01$
Residual attention	$0.71\pm 0.02$	$0.68\pm 0.02$	$0.85\pm 0.02$	$0.68\pm 0.03$	${\textbf {0.78}}\pm 0.02$	$0.86\pm 0.03$	$0.64\pm 0.04$	$0.74\pm 0.01$
Our model	${\textbf {0.79}}\pm 0.01$	${\textbf {0.75}}\pm 0.01$	$0.85\pm 0.02$	${\textbf {0.73}}\pm 0.01$	${\textbf {0.78}}\pm 0.01$	$0.89\pm 0.00$	${\textbf {0.66}}\pm 0.02$	${\textbf {0.78}}\pm 0.00$
No YOLO	$0.71\pm 0.01$	$0.67\pm 0.01$	$0.78\pm 0.02$	$0.70\pm 0.01$	$0.77\pm 0.01$	$0.90\pm 0.01$	$0.60\pm 0.02$	$0.73\pm 0.01$

For this metric, we considered erroneous the cases in which the expert gave a descriptor and the model did not, while for kappa, we only considered the cases where both the model and the expert gave a descriptor

**Table 5:** Agreement on the BI-RADS classification with the expert. Comparison of the BI-RADS categories assigned by the radiologist with the ones obtained by the multinomial logistic regression, receiving as input the descriptors of the different CNNs

	**Accuracy**		**Kappa**	
**Expert intercorrelation**	–		$0.47\pm 0.11$	
**Expert intracorrelation**	–		$0.75\pm 0.03$	
Model	cross-validation	testing	cross-validation	testing
VGG16	$0.55\pm 0.04$	$0.58\pm 0.02$	$0.45\pm 0.05$	$0.49\pm 0.03$
ResNet	$0.55\pm 0.07$	$0.58\pm 0.03$	$0.45\pm 0.09$	$0.49\pm 0.03$
DenseNet	$0.55\pm 0.06$	$0.56\pm 0.02$	$0.45\pm 0.08$	$0.46\pm 0.03$
RAD-DenseNet	$0.53\pm 0.05$	$0.56\pm 0.05$	$0.43\pm 0.06$	$0.46\pm 0.06$
MobileNet	$0.52\pm 0.06$	$0.55\pm 0.02$	$0.42\pm 0.07$	$0.45\pm 0.03$
Residual attention	$0.54\pm 0.06$	$0.56\pm 0.03$	$0.44\pm 0.07$	$0.46\pm 0.03$
Our model	${\textbf {0.61}}\pm 0.05$	${\textbf {0.60}}\pm 0.01$	${\textbf {0.53}}\pm 0.06$	${\textbf {0.52}}\pm 0.02$
No YOLO	$0.44\pm 0.06$	$0.56\pm 0.02$	$0.32\pm 0.08$	$0.46\pm 0.02$

**Table 6:** Confusion matrix for the shape descriptor. Comparison of our model and the residual attention model with the expert for the first repetition of cross-validation

	Our model				Residual attention				Total
	Oval	Round	Lobulated	Irregular	Oval	Round	Lobulated	Irregular	
Oval	**180**	10	10	22	**161**	21	20	20	222
Round	20	**14**	5	3	20	**14**	5	3	42
Lobulated	22	3	**11**	24	32	7	**7**	14	60
Irregular	8	2	11	**192**	13	4	17	**179**	213
Total	230	29	37	241	226	46	49	216	537

**Table 7:** Confusion matrix for the margin descriptor. Comparison of both our model and the residual attention model with the expert for the first repetition of cross-validation

	Our model					Residual attention					Total
	Circum.	Micro.	Non-defined	Angulated	Spiculated	Circum.	Micro.	Non-defined	Angulated	Spiculated	
Circum.	**253**	2	37	1	0	**253**	6	32	2	0	293
Micro.	1	**0**	21	0	3	5	**2**	13	3	2	25
Non-defined	21	6	**119**	0	12	28	6	**98**	6	20	158
Angulated	5	1	17	**0**	1	5	5	13	**0**	1	24
Spiculated	0	0	19	0	**16**	0	2	19	0	14	35
Total	280	9	213	1	32	291	21	175	11	37	535

**Table 8:** Confusion matrix for the orientation descriptor. Comparison of both our model and the residual attention model with the expert for the first repetition of cross-validation

	Our model		Total	Residual attention		Total
	Parallel	Antiparallel		Parallel	Antiparallel	
Parallel	**344**	22	366	**322**	27	349
Antiparallel	30	**57**	87	20	**67**	87
Total	374	79	453	342	94	436

**Table 9:** Confusion matrix for the echogenicity descriptor. Comparison of both our model and the residual attention model with the expert for the first repetition of cross-validation

	Our model				Residual attention				Total
	Anechoic	Hypoechoic	Heterogeneous	Isoechoic	Anechoic	Hypoechoic	Heterogeneous	Isoechoic	
Anechoic	**94**	13	4	1	**93**	15	4	0	112
Hypoechoic	5	**176**	53	3	6	**160**	66	5	237
Heterogeneous	7	52	**109**	1	5	60	**101**	3	169
Isoechoic	0	11	4	**1**	1	5	6	**4**	16
Total	106	252	170	6	105	240	177	12	537

**Table 10:** Confusion matrix for the posterior feature descriptor. Comparison of both our model and the residual attention model with the expert for the first repetition of cross-validation

	Our model			Residual attention			Total
	Refuerzo	Sin cambios	Sombra	Refuerzo	Sin cambios	Sombra	
Refuerzo	**254**	38	12	**261**	35	8	304
Sin cambios	48	**57**	14	42	**59**	18	119
Sombra	10	12	**61**	16	8	**59**	83
Total	312	107	87	319	102	85	506

**Table 11:** Confusion matrix for the echogenic halo descriptor. Comparison of both our model and the residual attention model with the expert for the first repetition of cross-validation

	Our model		Residual attention		Total
	Halo	No halo	Halo	No halo	
Halo	**120**	37	**110**	47	157
No halo	41	**329**	36	**334**	370
Total	161	366	146	381	527

**Table 12:** Confusion matrix for the suggestivity. Comparison of both our model and the residual attention model with the expert for the first repetition of cross-validation. The labels “simple c.”, “complex c.”, and “fibro.” are abbreviations for simple cyst, complex cyst, and fibroadenoma

	Our model			total	Residual attention			Total
	simple c.	complex c.	fibro.		simple c.	complex c.	fibro.	
Simple c.	**89**	9	5	103	**76**	12	3	91
Complex c.	9	**16**	1	26	12	**7**	6	25
Fibro.	0	4	**21**	25	0	5	**23**	28
Total	98	29	27	154	88	24	32	147

**Table 13:** Confusion matrix for the BI-RADS classification. Comparison of both our model and the residual attention model with the expert for the first repetition of cross-validation

	Our model						Residual attention						Total
	2	3	4A	4B	4C	5	2	3	4A	4B	4C	5	
2	**96**	8	11	1	0	0	**85**	11	18	2	0	0	116
3	7	**45**	24	5	4	0	9	**39**	28	2	5	2	85
4A	8	28	**54**	17	6	0	12	23	**52**	15	11	0	113
4B	1	4	13	**26**	20	1	0	3	19	**22**	19	2	65
4C	1	2	6	13	**48**	22	2	3	9	15	**37**	26	92
5	0	0	1	1	20	**47**	0	0	0	2	15	**52**	69
Total	113	87	109	63	98	70	108	79	126	58	87	82	540

**Table 14:** Tukey post-hoc test in the test experiment

	VGG16	ResNet	DenseNet	Rad-DenseNet	MobileNet	Residual attention
**Accuracy**						
Our model	0.5755	**0.0310**	0.3012	**0.0310**	**0.0095**	**0.0142**
Model without descriptors	0.3840	**0.0142**	0.1726	**0.0142**	**0.0041**	**0.0063**
**F1**						
Our model	0.6401	**0.0419**	0.4899	**0.0130**	**0.0236**	**0.0316**
Model without descriptors	0.1915	**0.0051**	0.1201	**0.0014**	**0.0027**	**0.0037**

The only other statistically significant differences between the models were found in recall, between the “model without descriptors” and Rad-DenseNet, with $p=0.0026$, and in precision, between our model and MobileNet, with $p=0.0235$

## Footnotes

1 https://www.cancer.org/cancer/breast-cancer/understanding-a-breast-cancer-diagnosis/breast-cancer-survival-rates.html

2 https://www.ultrasoundcases.info/
